# Possible effect of landscape design on IgE recognition profiles of two generations revealed with micro‐arrayed allergens

**DOI:** 10.1111/all.13169

**Published:** 2017-05-11

**Authors:** V. Garib, E. Wollmann, G. Djambekova, P. Lemell, M. Kmenta, U. Berger, P. Zieglmayer, R. Valenta

**Affiliations:** ^1^ Division of Immunopathology Department of Pathophysiology and Allergy Research Center of Pathophysiology Infectiology and Immunology Medical University of Vienna Vienna Austria; ^2^ Specialized Scientific‐Practical Center for Therapy and Medical Rehabilitation (RSSPMC T&R) Tashkent Uzbekistan; ^3^ Vienna Challenge Chamber Vienna Austria; ^4^ Department of Oto‐Rhino‐Laryngology Medical University of Vienna Vienna Austria

**Keywords:** allergen, component‐resolved diagnosis, IgE profiles, landscape design, molecular allergology

## Abstract

The aim of this study was to investigate possible effects of landscape design on the IgE sensitization profile toward inhalant allergens in patients with respiratory allergy from Uzbekistan where green areas have been changed during the last two decades by a State program. Sera from two different generations of Uzbek (n=58) and, for control purposes, from two generations of Austrian (n=58) patients were analyzed for IgE reactivity to 112 different micro‐arrayed allergen molecules by ImmunoCAP ISAC technology. Changes in molecular IgE sensitization profiles to pollen allergens in the young vs the middle‐aged Uzbek population were associated with replanting, whereas those in the Vienna populations reflected natural changes in plant growth. Our data indicate that anthropologic as well as natural changes in the biome may have effects on IgE sensitization profiles already from one to another generation.

## 1 Introduction

Uzbekistan's vegetation originally consisted of approximately 492 cultivated plants from 79 families and 577 wild herbs. Due to economic growth in the past two decades, anthropogenic landscaping took place in Tashkent, the capital of Uzbekistan. Within the city, vegetation and flora have been actively reorganized by eradicating wild roadside weeds, and green carpets of decorative grasses, such as ryegrass and Bermuda grass, were introduced into the city. Currently, green areas occupy 35% of the city, and green space available for each resident of Tashkent rose up from 21 m^2^ to almost 69 m^2^, a more than threefold increase during the last twenty years.^1^ The planting of trees such as oak, chestnut, pine, spruce, linden, Japanese cedar, and cotton trees also led to improved air quality.

It is well established that allergic sensitization profiles depend on the environment and habits of given populations. In a very elegant study, Moverare and colleagues demonstrated that recombinant birch pollen allergen Bet v 1 could be used as a marker allergen to reveal that the lower incidence of birch pollen allergy in southern parts of Europe reflected lower exposure to birch pollen, whereas sensitization to cross‐reactive allergens due to exposure to other plants increased.^2^ Likewise, molecular diagnosis with recombinant allergens revealed different sensitization profiles regarding olive and grass pollen allergens in different regions of Spain as well as unique sensitizations profiles in Africa and in the Asia–Pacific area.^3^, ^4^, ^5^ Furthermore, there are major studies ongoing using micro‐arrayed allergens to study the molecular recognition profiles in different parts of Europe.^6^ However, so far no attempt has been made to investigate whether changes in the environment through anthropologic transformation of the biome may have an effect on allergic sensitization profiles in a population. To study this question, we determined the prevalence and patterns of IgE‐mediated sensitization toward a broad panel of pollen allergen molecules in sera from two generations of patients with respiratory allergy from Tashkent using micro‐arrayed allergens.

## 2 Methods

One hundred and twenty patients with respiratory allergy referred to the Allergy and Respiratory Unit of Republic's Specialized Scientific‐Practical Center for Therapy and Medical Rehabilitation (RSSPMC T&R), Tashkent, Uzbekistan, were studied. Patients were interviewed and included in the study only if

- they were born and always lived in Tashkent.
- they were of Uzbek nationality for three or more generations.
- they suffered from respiratory pollen allergy.
- they had not undergone allergen‐specific immunotherapy (AIT) with pollen/plant allergens.

**Figure 1:**
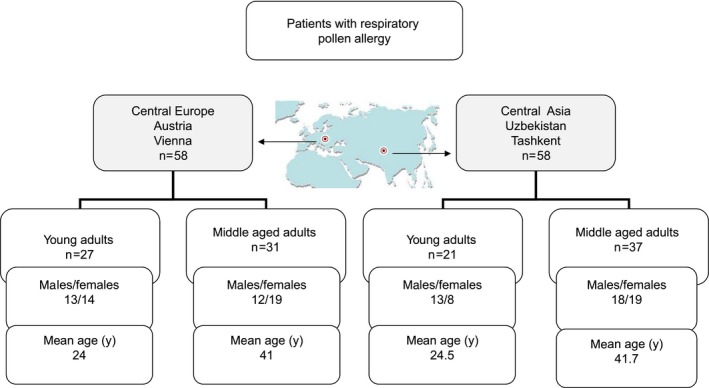
Characterization of the studied patients. [Colour figure can be viewed at wileyonlinelibrary.com]

Patients fulfilling these inclusion criteria (n=58) were divided into two groups according to anthropological periodization and landscape program implementation in Tashkent: a group of young adults (18‐28 years; mean age 24.5 years; n=21) and a group of middle‐aged adults (29‐49 years; mean age: 41.7 years; n=37) (Figure 1). There were no relevant differences regarding lifestyle (ie, housing, diet). The only drastic change in living habits during the last twenty years was that families started to keep cats as pets inside their houses/apartments.

For control purposes, we investigated a group of 130 patients suffering from respiratory pollen allergy from Vienna, Austria. In Vienna, no program‐driven landscaping had taken place. Records of natural changes in the plant landscape in Vienna City were available from the Forestry and Urban Agriculture Office of Vienna (Forstamt und Landwirtschaftsbetrieb der Stadt Wien) for the period 1993‐2015. Tree pollen loads in Vienna, including *Fraxinus* and total *Oleaceae*,* Betulaceae,* and *Cupressaceae/Taxaceae* pollen, were measured by the Aerobiologic Section of the ENT Department during 1993‐2015 using a Hirst‐type volumetric pollen and spore trap (Burkard Manufacturing, Rickmansworth, UK) followed by a microscopic analysis.

Patients (n=58) from Vienna were included if they were born and always lived in Vienna and were Austrian for more than three generations and never had AIT with pollen/plant allergens. These patients were also grouped in young (18‐28 years; mean age 24 years; n=27) and middle‐aged (29‐59 years; mean age 41 years; n=31) adults (Figure 1). The control groups thus showed a similar composition regarding age and were also similar regarding the gender balance as compared to the Uzbek groups (Figure 1). Neither in the Uzbek nor in the Austrian young groups, subjects below 18 years were included because it has been shown that children up to the age of 12 years can acquire additional sensitizations whereas in adult patients IgE reactivity profiles remain constant.^7^, ^8^, ^9^ Permission of the local ethics committees was obtained, and written informed consent was obtained from the patients. Allergen‐specific IgE antibodies were measured in anonymized serum samples using the ImmunoCAP ISAC micro‐array technology containing 112 allergens according to the manufacturer's recommendations (Thermo Fisher, Uppsala, Sweden) in the Department of Pathophysiology and Allergy Research of the Medical University of Vienna with permission from the Ethics Committee of the Medical University of Vienna (EK565/2007). Distribution of IgE sensitization to allergen molecules was analyzed using IBM SPSS Statistics 20 and Microsoft Excel.

## 3 Results and Discussion

**Figure 2:**
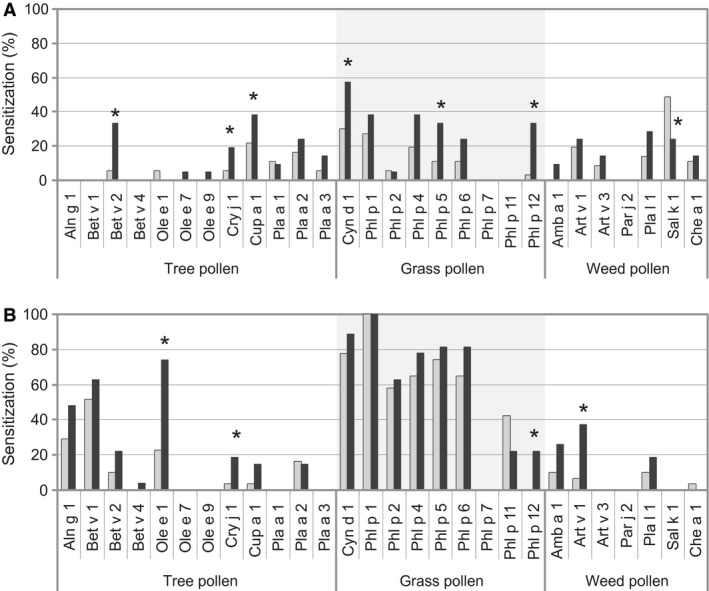
Frequencies of IgE recognition of respiratory allergens in young (black bars) and in middle‐aged adult (gray bars) Tashkent (A) and Viennese (B) patients with respiratory pollen allergy. Allergen molecules of tree, grass, and weed pollen are shown on the x‐axes, and frequencies of IgE reactivities are displayed in percentages of the patients (y‐axes), *P<0.05.

The analysis of the allergen IgE recognition frequencies in the young and middle‐aged patients from Tashkent revealed striking and highly significant differences. nSal k 1 from saltwort (Salsola kali) was the most prevalent weed allergen recognized by the middle‐aged population with a sensitization rate of 48.6% in patients older than 30 years. This fits to reports from countries which are close to Tashkent. Also for southern Europe, western United States, and semidesert areas of Saudi Arabia, Kuwait, and Iran, saltwort represents a major allergen source which besides the major allergen Sal k 1 contains also additional other allergens.^10^ In contrast to the middle‐aged population, we found a significantly lower IgE sensitization rate to Sal k 1 in the young population (ie, 23.8%) (Figure 2), which may be explained by the eradication of saltwort through the reorganization of the landscape in the last 20 years. Consistent with the landscape reorganization, we found significant increases in IgE sensitizations toward the grass pollen allergens Cyn d 1 and Phl p 5 as well as to the allergens of cypress Cry j 1 and cedar Cup a 1 (Figure 2). The increase in IgE recognition of profilins (Bet v 2, Phl p 12, Mer a 1) may well be explained by replanting with ornamental plants and eventually also by increased grass pollen exposure. The sensitization rates to the mugwort allergens showed no significant differences between the two age groups from Tashkent.

Unlike in Tashkent where the frequencies of IgE reactivities to grass pollen allergens increased due to replanting, the frequencies of IgE reactivities to grass pollen allergens did not change in Vienna (Figure 2). Likewise, sensitizations to the major tree pollen allergens from birch, Bet v 1, and alder, Aln g 1, showed no significant changes in Vienna. However, the frequencies of IgE reactivities to certain allergens increased in the young Vienna population significantly, such as to Ole e 1, which is a marker for ash sensitization^11^; to Cry j 1, a marker for allergy to *Cupressaceae*; and to the major mugwort allergen Art v 1. We therefore studied data regarding changes in natural growth of plants in the Vienna area and found that the volume of ash trees has almost doubled in Vienna when comparing the years 1993‐2002 with the period 2003‐2015. Likewise, the volume of Douglas trees doubled in the period 2003‐2015, whereas birch and alder volumes did not increase. Pollen count measurements showed that there were strong increases in ash and cypress pollen in the latter period, whereas birch pollen exposure showed no relevant difference (Table S1A‐C). Regarding mugwort and ragweed, it is known that weed pollen exposure is on a continuous rise in Vienna due to climate changes and migration of weeds from Hungary and Eastern Europe toward Vienna.^12^, ^13^

The changes in molecular sensitization profiles found in the younger generations of the Uzbek and Austrian pollen‐allergic patients were associated with the changes in landscape due to the governmental replanting in Uzbekistan and natural changes in vegetation in Austria. One may therefore hypothesize that changes in vegetation occurring through coordinated programs and/or natural changes may have effects on the IgE sensitization profiles in patients with respiratory allergies within one generation (ie, only 20 years). A limitation of our study is the relatively low number of patients, but the obtained results were clear and consistent. For example, the increase in grass in Tashkent was reflected in an increase of sensitization (rates and IgE levels) towards several different major grass pollen allergens, and changes regarding sensitization to cross‐reactive allergens (eg, profilin) were consistent (Figure 2; Supplemental Table 2). Another limitation may be that IgE reactivities were measured using the micro‐array technology, but it has been demonstrated that ImmunoCAP ISAC technology is as sensitive as ImmunoCAP technology.^6^ Should further studies corroborate our findings, one may speculate that it is possible to influence allergic sensitization profiles by controlled changes in the environment for the prevention of certain allergies.

## Conflict of Interest

Rudolf Valenta has received research grants from Biomay AG, Vienna, Austria; Thermo Fisher, Uppsala, Sweden; and Fresenius Medical Care, Bad Homburg, Germany, and serves as a consultant to these companies. The other authors have no conflict of interest to declare.

## Author Contributions

VG performed experiments, analyzed the data, wrote the manuscript, and read the manuscript; EW and GD performed experiments, analyzed the data, and read the manuscript; RV designed and supervised experiments, analyzed the data, wrote the manuscript, and read the manuscript; MK and UB provided and analyzed the data and read the manuscript; and PL and PZ analyzed the data and read the manuscript.
