# Supplementary material for: Possible effect of landscape design on IgE recognition profiles of two generations revealed with micro‐arrayed allergens
Source: Allergy. 2017 May 11;72(10):1579–82. doi: 10.1111/all.13169 (PMC5638056; doi:10.1111/all.13169)
Supplement: Supplementary file 1 [file ALL-72-1579-s001.doc]

Supplemental Table 1

A

|  |  |  |  |  |  |
| --- | --- | --- | --- | --- | --- |
|  | Annual total | Annual total | Peak | Days >50 | >100 |
| Year | *Fraxinus* pollen | *Oleacea* pollen | value | grains/m³ | grains/m³ |
|  |  |  |  |  |  |
| 1993 | 1192 | 1213 | 130 | 7 | 3 |
| 1994 | 2821 | 2854 | 530 | 15 | 6 |
| 1995 | 2595 | 2626 | 476 | 13 | 8 |
| 1996 | 522 | 523 | 253 | 2 | 2 |
| 1997 | 570 | 580 | 51 | 1 | 0 |
| 1998 | 1658 | 1660 | 190 | 9 | 4 |
| 1999 | 5289 | 5290 | 613 | 17 | 13 |
| 2000 | 1633 | 1653 | 153 | 12 | 3 |
| 2001 | 2318 | 2327 | 271 | 12 | 8 |
| 2002 | 3517 | 3548 | 361 | 18 | 11 |
| mean | **2212** | **2227** | **303** | **11** | **6** |
|  |  |  |  |  |  |
| 2003 | 8014 | 8033 | 1758 | 22 | 17 |
| 2004 | 2973 | 2983 | 420 | 17 | 10 |
| 2005 | 9952 | 9975 | 2232 | 16 | 11 |
| 2006 | 9073 | 9083 | 1195 | 19 | 17 |
| 2007 | 4192 | 4212 | 534 | 16 | 12 |
| 2008 | 7114 | 7134 | 871 | 35 | 22 |
| 2009 | 3569 | 3595 | 789 | 12 | 8 |
| 2010 | 2952 | 2986 | 372 | 17 | 8 |
| 2011 | 7882 | 7904 | 1505 | 16 | 14 |
| 2012 | 2601 | 2620 | 353 | 13 | 8 |
| 2013 | 7845 | 7883 | 2402 | 15 | 14 |
| 2014 | 1570 | 1596 | 280 | 8 | 5 |
| 2015 | 7720 | 7752 | 983 | 20 | 16 |
| mean | **5804** | **5827** | **1053** | **17** | **12** |

B

|  |  |  |  |  |
| --- | --- | --- | --- | --- |
| Year | Annual total | Peak | Days >50 | >100 |
|  | *Betulaceae* pollen | value | grains/m³ | grains/m³ |
|  |  |  |  |  |
| 1993 | 14048 | 2672 | 34 | 29 |
| 1994 | 17856 | 1803 | 64 | 40 |
| 1995 | 10057 | 813 | 48 | 32 |
| 1996 | 6428 | 2660 | 19 | 11 |
| 1997 | 4343 | 374 | 27 | 15 |
| 1998 | 6898 | 1002 | 29 | 18 |
| 1999 | 5983 | 574 | 30 | 18 |
| 2000 | 5850 | 435 | 23 | 20 |
| 2001 | 10911 | 1350 | 55 | 34 |
| 2002 | 9550 | 938 | 48 | 25 |
| mean | **9192** | **1262** | **38** | **24** |
|  |  |  |  |  |
| 2003 | 17469 | 1281 | 48 | 42 |
| 2004 | 18646 | 1937 | 49 | 37 |
| 2005 | 7126 | 653 | 31 | 17 |
| 2006 | 20363 | 2396 | 42 | 36 |
| 2007 | 8713 | 812 | 40 | 25 |
| 2008 | 21851 | 1656 | 67 | 42 |
| 2009 | 5396 | 756 | 23 | 13 |
| 2010 | 16510 | 1555 | 40 | 35 |
| 2011 | 8540 | 658 | 37 | 27 |
| 2012 | 11545 | 1700 | 33 | 19 |
| 2013 | 10627 | 988 | 33 | 22 |
| 2014 | 11142 | 1713 | 46 | 31 |
| 2015 | 6836 | 754 | 25 | 14 |
| mean | **12674** | **1297** | **40** | **28** |
|  |  |  |  |  |

C

|  |  |  |  |  |
| --- | --- | --- | --- | --- |
| Year | Annual total | Peak | Days >50 | >100 |
|  | *Cupress/Taxaceae* pollen | value | grains/m³ | grains/m³ |
|  |  |  |  |  |
| 1993 | 1800 | 159 | 15 | 3 |
| 1994 | 2794 | 511 | 14 | 6 |
| 1995 | 3056 | 497 | 13 | 7 |
| 1996 | 3116 | 751 | 10 | 9 |
| 1997 | 2122 | 482 | 11 | 6 |
| 1998 | 2226 | 509 | 10 | 5 |
| 1999 | 3734 | 517 | 19 | 10 |
| 2000 | 2034 | 292 | 11 | 5 |
| 2001 | 3119 | 501 | 15 | 8 |
| 2002 | 3726 | 671 | 16 | 7 |
| mean | **2773** | **489** | **13** | **7** |
|  |  |  |  |  |
| 2003 | 23779 | 5896 | 28 | 22 |
| 2004 | 14056 | 3000 | 25 | 12 |
| 2005 | 17659 | 4787 | 25 | 18 |
| 2006 | 23897 | 6470 | 30 | 21 |
| 2007 | 17982 | 2702 | 44 | 27 |
| 2008 | 17154 | 8140 | 22 | 18 |
| 2009 | 16916 | 4094 | 21 | 12 |
| 2010 | 16094 | 5451 | 19 | 13 |
| 2011 | 13614 | 2870 | 25 | 22 |
| 2012 | 13258 | 2147 | 24 | 20 |
| 2013 | 16329 | 3291 | 32 | 23 |
| 2014 | 15769 | 2954 | 24 | 20 |
| 2015 | 20229 | 2441 | 37 | 22 |
| mean | **17441** | **4173** | **27** | **19** |
|  |  |  |  |  |
