# Supplementary figures and images for: Possible effect of landscape design on IgE recognition profiles of two generations revealed with micro‐arrayed allergens
Source: Allergy. 2017 May 11;72(10):1579–82. doi: 10.1111/all.13169 (PMC5638056; doi:10.1111/all.13169)

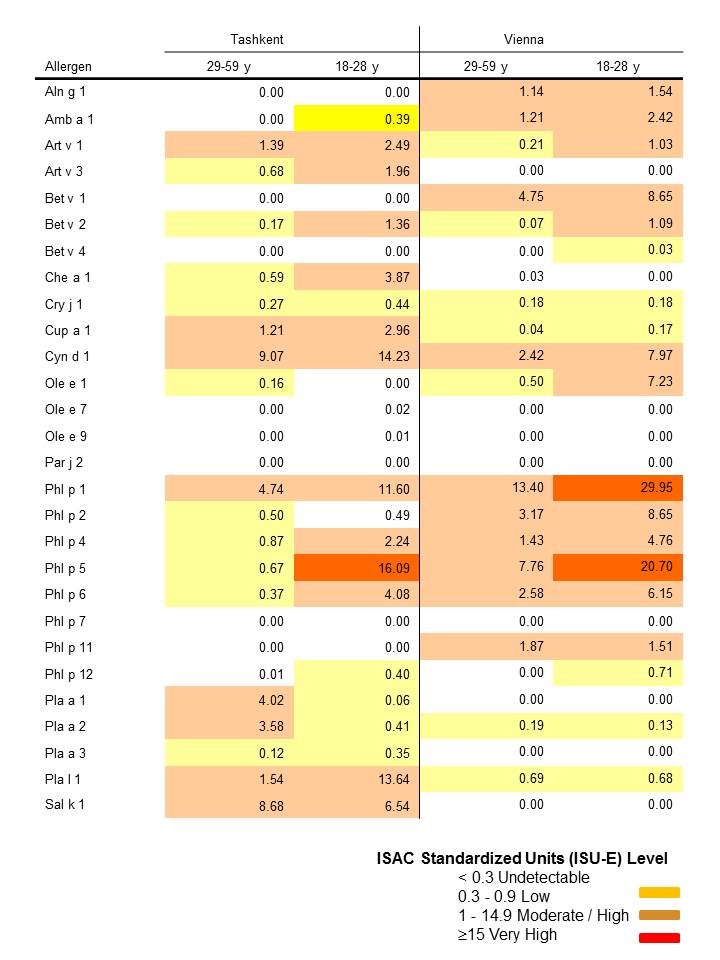

Supplement: Supplementary file 2 [file ALL-72-1579-s002.jpg]
